# MSA Mimic? Rare Occurrence of Anti-Hu Autonomic Failure and Thymoma in a Patient with Parkinsonism: Case Report and Literature Review

**DOI:** 10.3389/fnins.2018.00017

**Published:** 2018-01-24

**Authors:** Vito A. G. Ricigliano, Barbara Fossati, Lorenzo Saraceno, Michele Cavalli, Elena Bazzigaluppi, Giovanni Meola

**Affiliations:** ^1^Department of Biomedical Sciences for Health, University of Milan, IRCCS Policlinico San Donato, Milan, Italy; ^2^Autoimmunity Service, Laboratory Medicine, San Raffaele Hospital, Milan, Italy; ^3^Department of Neurology, IRCCS Policlinico San Donato, Milan, Italy

**Keywords:** paraneoplastic syndrome, dysautonomia, anti-Hu, Parkinson, MSA

## Abstract

Thymoma is a tumor originating from thymic gland, frequently manifesting with paraneoplastic neurological disorders. Its association with paraneoplastic dysautonomia is relatively uncommon. Here, we describe the challenging case of a 71 year-old female who developed subacute autonomic failure with digestive pseudo-obstruction, dysphagia, urinary tract dysfunction and orthostatic hypotension complicating an underlying extrapyramidal syndrome that had started 3 months before hospital admission. Autonomic symptoms had 2-month course and acutely worsened just before and during hospitalization. Combination of severe dysautonomia and parkinsonism mimicked rapidly progressing multiple system atrophy. However, diagnostic exams showed thymic tumor with positive anti-Hu antibodies on both serum and cerebrospinal fluid. Complete response of dysautonomia to immunoglobulins followed by thymectomy confirmed the diagnosis of anti-Hu-related paraneoplastic neurological syndrome. With regards to extrapyramidal symptoms, despite previous descriptions of paraneoplastic parkinsonism caused by other antineuronal antibodies, in our case no relation between anti-Hu and parkinsonism could be identified. A literature review of published reports describing anti-Hu positivity in thymic neoplasms highlighted that a definite autonomic disease due to anti-Hu antibodies is extremely rare in patients with thymoma but without myasthenia gravis, with only one case published so far.

## Background

Thymic neoplasms are mediastinal tumors often associated to the production of several autoantibodies and heterogeneous paraneoplastic neurological syndromes (PNS), the most frequent being myasthenia gravis (MG) (Barua et al., 2012). In these cases, association with anti-Hu (also called ANNA-1) antibodies is very rare and has been described in only 9 individuals to date (Vernino et al., 2002; Simonelli et al., 2009; Okamoto et al., 2011; Barua et al., 2012; Yang et al., 2014), presenting in almost half of patients with painful or sensory neuropathy, or with MG and positive anti-neuromuscular junction antibodies. Clinical dysautonomia has been reported in 2 subjects and manifested as gastroparesis and intestinal pseudo-obstruction with other accompanying symptoms like fixed dilated pupils or dry eyes and mouth.

We present the case of a female patient with thymoma but without MG, who developed severe subacute anti-Hu-related dysautonomia that worsened a concurrent extrapyramidal syndrome. Paraneoplastic disease had optimal response to therapy, with complete resolution after combination of intravenous immunoglobulins and thymectomy.

## Case presentation

A previously healthy 71 year-old female, nonsmoker, presented to the Emergency Department complaining of marked slowness of thought, excessive daytime sleepiness, generalized weakness with reduced motor ability, severe constipation, urinary retention, episodic dysphagia and sialorrhoea. More in detail, the patient and her relatives traced back the insidious onset of motor impairment, slowness of thought and sialorrhoea to 3 months before. Bowel disorder presented 1 month later and progressed over time. Bladder dysfunction appeared 15 days before admission and acutely worsened together with cognitive-motor slowing and enteric symptoms soon after a colonoscopy performed to investigate intestinal hypomotility. No adverse or long-lasting effects of sedative drugs used during the procedure were reported. Neurological examination showed hypomimia, bradyphrenia, severe bradykinesia, micrographia, bilateral limb rigidity with minimal left side prevalence and mild rest tremor, reduced arm swing while walking, consistent with a diagnosis of extrapyramidal syndrome accompanied by significant dysautonomia. No vertical eye movement dysfunction or history of repeated falls could be detected. Mini-mental state examination (MMSE) showed normal results. Based on combination of symptoms, primary diagnostic hypothesis was multiple system atrophy (MSA), but acute-subacute autonomic worsening looked suspicious. Patient was admitted to our Neurological Department for further investigations. On day three after hospitalization, constipation dramatically worsened featuring intestinal pseudo-obstruction resistant to any laxative medications and an episode of acute urinary retention led to bladder catheterization. Moreover, she developed orthostatic hypotension with blood pressure values of 125/80 mmHg in supine position and 95/70 mmHg within 3 min of standing (Freeman et al., 2011). Hypotension was attributed to the dysautonomic syndrome but, to note, no specific autonomic tests to confirm its neurogenic nature were performed.

Routine chest X ray showed irregular mediastinal widening with lobulated aspect, better characterized by contrast chest computed tomography (CT) scan as possible thymoma/thymic carcinoma versus germ cell tumor or lymphoma. Given this finding, patient underwent abdomen CT scan and total body positron emission tomography (PET) that excluded systemic dissemination of disease. Neoplastic involvement of the brain or the meninges was ruled out by means of contrast-enhanced brain magnetic resonance imaging (MRI) and cerebrospinal fluid (CSF) cell analysis, which was completely normal. Routine blood tests, autoimmune screening (including anti-nuclear antibodies), peripheral blood smear and oncomarkers were negative. Thyroid exams showed mild hypothyroidism, with highly positive anti-thyroid antibodies, but clinical presentation and normal electroencephalogram (EEG) did not fulfill diagnostic criteria for Hashimoto's encephalopathy (Graus et al., 2016) as a cause for the severe slowness of thought.

**Figure 1:**
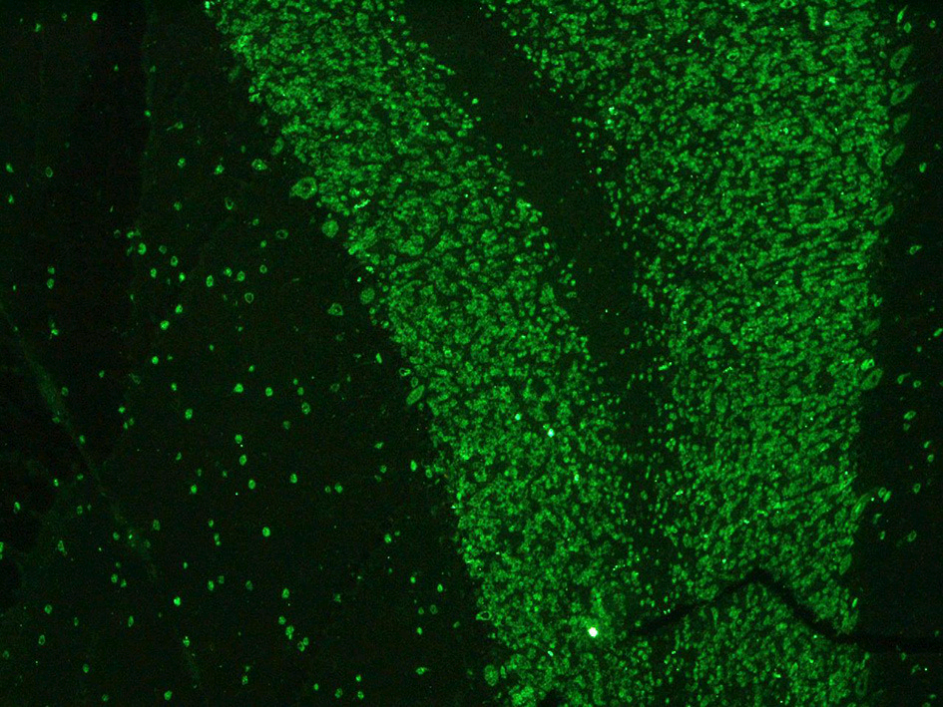
Positive serum anti-Hu staining on monkey cerebellum commercial sections.

**Figure 2:**
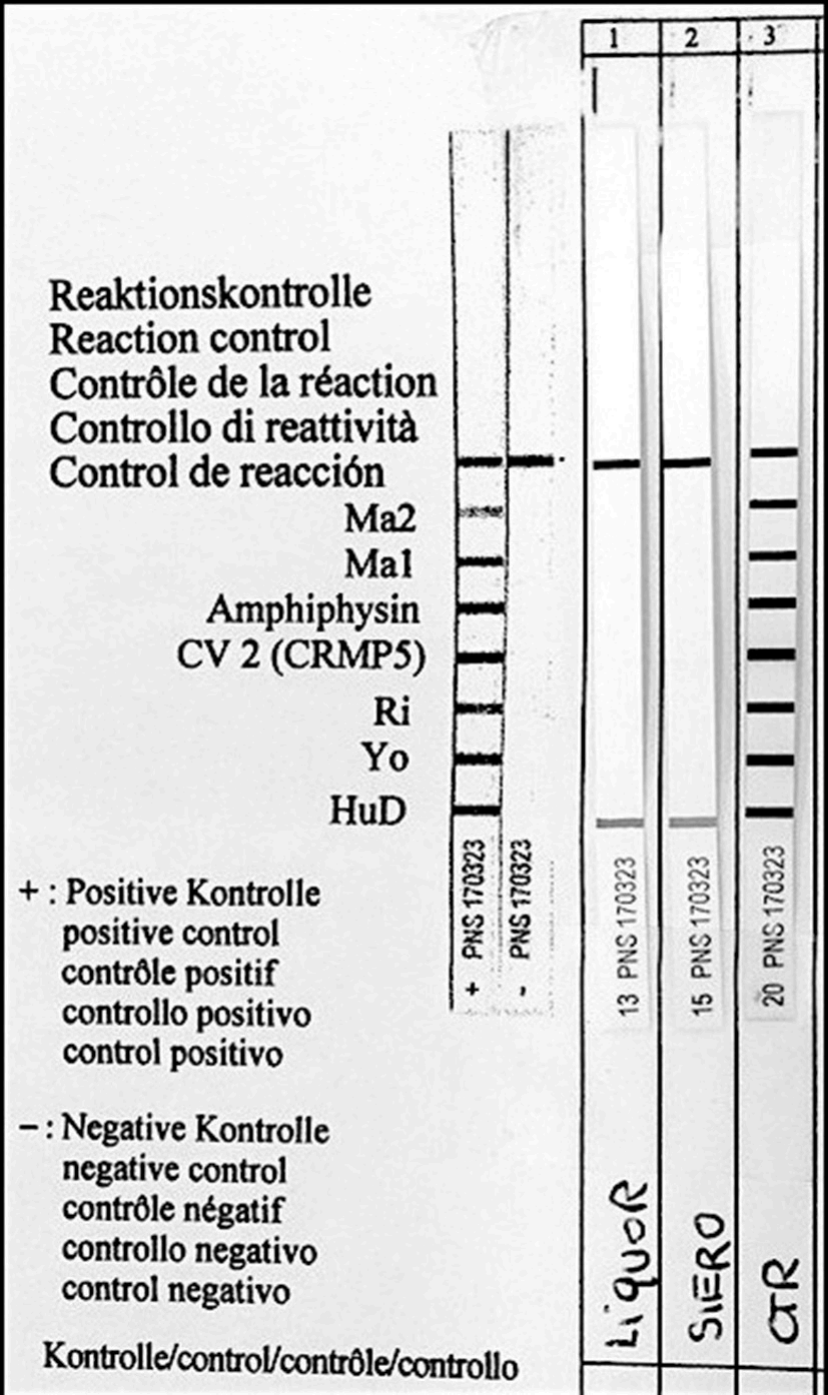
Identification of serum anti-Hu antibodies (Hu-D) by Western blot analysis. Liquor, CSF; Siero, serum; CTR, positive control.

Paraneoplastic etiology for both dysautonomia and extrapyramidal symptoms, given their subacute presentation and strict temporal association, was therefore hypotesized. Tests for anti-Hu, Ri, Yo, Ma2, CV2/CRMP5, LGI1, CaspR2, NMDAR, and GAD were performed on serum and CSF and positive anti-Hu antibodies through indirect immunoflurescence test on monkey cerebellum commercial sections (Euroimmun®), confirmed with immunoblotting (Ravo Diagnostika®) were found in both conditions (Figures 1, 2). Positive immunofluorescence test results were also found after adding patient's anti-Hu on human myoenteric plexus commercial sections (image not shown). Notably, anti-NMDAR, GAD, CV2/CRMP5, and Ri antibodies, which have been previously reported to be associated with extrapyramidal syndromes (Tada et al., 2004; Pittock et al., 2006; Zandi et al., 2015; Mehta et al., 2016; Nabil et al., 2017), were absent.

Transthoracic needle biopsy of the mass confirmed the diagnosis of thymoma. Given the histological finding and history of episodic dysphagia, despite the absence of clinical MG, patient underwent repetitive motor nerve stimulation and anti-neuromuscular junction antibody (AchR, MuSK, calcium channel) dosage, both with negative results.

Therefore, based on recommended diagnostic criteria for PNS (Graus et al., 2004), patient was diagnosed with severe paraneoplastic dysautonomia due to positive anti-Hu antibodies (definite PNS with classical symptoms) accompanied by unrelated extrapyramidal syndrome. She underwent Movement Disorder Society-Unified Parkinson's Disease Rating Scale (MDS-UPDRS) evaluation, obtaining a total score of 105, and was started with L-dopa/carbidopa 200/50 mg half tablet 4 times a day, with minimal motor improvement (20% reduction in MDS-UPDRS score, possibly due to underlying malabsorption) and no change (either worsening—as it can sometimes be observed with L-dopa—or reduction) in autonomic symptoms. Treatment with intravenous immunoglobulins 400 mg/kg body weight daily for 5 days was attempted on day 30 after admission. Soon after therapy conclusion, autonomic manifestations dramatically improved, with resolution of intestinal pseudo-obstruction together with partial reduction of urinary retention and orthostatic hypotension. To note, cognitive and motor symptoms did not show any further modifications. 15 days later, the patient was transferred to Thoracic Surgery Unit for tumor removal, which determined complete normalization of dysphagia, sialorrhoea, enteric, and urinary symptoms. Histological examination of the mass confirmed a pT2N0 thymoma (modified Masaoka clinical staging 2b; Koga et al., 1994). Since tumor margins were not disease-free and thymoma was partially adherent to pericardium, radiotherapy (RT) with cumulative dose of 54 Gray (Gy) was performed within 3 months after surgery.

Complete response of autonomic problems to the combination of immunoglobulins and surgery allowed us to rule out diagnosis of MSA with predominant parkinsonism (MSA-P, based on 2008 consensus criteria; Gilman et al., 2008), which, as stated above, had been taken into account as possible differential diagnosis.

Later on, brain dopamine transporter single photon emission computed tomography (DaT-SPECT) imaging demonstrated asymmetric reduction in presynaptic dopamine uptake in the basal ganglia. At 6-month follow-up evaluation, MDS-UPDRS score was 70, no signs or symptoms of dysautonomia could be identified and urodynamic study showed normal results, thus further excluding a diagnosis of MSA-P.

## Discussion

Autonomic disturbances are frequent in Parkinson disease and atypical parkinsonisms such as MSA-P, where autonomic failure may lead to significant orthostatic hypotension, genitourinary problems with urinary incontinence or incomplete bladder emptying and erectile dysfunction in males (Gilman et al., 2008; Fanciulli and Wenning, 2015). Early and severe dysautonomia is a key feature of disease, usually showing progression over time due to neurodegenerative changes affecting the central autonomic nervous system, including hypothalamus, nucleus ambiguus, dorsal nucleus of the vagus, intermediolateral columns of the spinal cord and Onuf nucleus (Benarroch, 1993). In the case described above, we observed a severe autonomic disorder complicating an underlying incipient extrapyramydal syndrome. However, acute course, with rapidly worsening constipation, acute urinary retention and dysphagia seemed hardly compatible with parkinsonism-related dysautonomia. Moreover, brain MRI for the study of pathologic changes in putamen, middle cerebellar peduncles, pons and cerebellum did not show features of MSA-P (Gilman et al., 2008). Given the presence of thymoma, a possible tumor-related specific involvement of autonomic nerves was suspected. Positive anti-Hu antibodies on both serum and CSF corroborated our hypothesis. Indeed, ANNA-1 are known to produce definite PNS syndrome with chronic gastrointestinal pseudo-obstruction or, less frequently, acute pandysautonomia (Antoine and Camdessanché, 2017). Paraneoplastic etiology of autonomic dysfunction was further reinforced by the positive therapeutic effect of immunoglobulins plus thoracic surgery, which weakened the alternative diagnosis of MSA-P with incomplete response to L-dopa (this feature being more likely due to intestinal malabsorption rather than “true” drug unresponsiveness, which is a mandatory diagnostic criterion for probable MSA-P; Gilman et al., 2008; Fanciulli and Wenning, 2015).

As regards to parkinsonism, the paraneoplastic form is a well-known, despite uncommon, entity whose first reports were described almost 30 years ago, with the finding of degeneration of substantia nigra in a patient with infiltrating ductal breast carcinoma (Golbe et al., 1989). Since then, several different autoantibodies have been associated to the development of clinically heterogeneous extrapyramidal syndromes, presenting with dystonia, dyskinesia, corea or other motor symptoms (Tada et al., 2004; Pittock et al., 2006; Zandi et al., 2015; Dash et al., 2016; Mehta et al., 2016; Nabil et al., 2017). However, to our knowledge, no clear evidence of causal relation between anti-Hu antibodies and parkinsonism has been reported so far. Even in our case, clinical features and absence of therapeutic effects of immunoglobulins plus thymectomy on parkinsonism do not fulfill criteria for a paraneoplastic genesis of the observed extrapyramidalism (Graus et al., 2004).

Anti-Hu antibodies are rarely associated with thymoma. In a review of 200 patients with ANNA-1 positivity and PNS, cancer was found in 83% of them, with most subjects having small cell lung cancer and none thymic neoplasms (Graus et al., 2001). Autonomic system involvement was identified in 19 cases with predominant neuropathy. Furthermore, retrospective screening of ANNA-1 positivity in 172 individuals with thymoma calculated an overall frequency of 3%, corresponding to 5 patients (Vernino et al., 2002), whose data are also reported in a more recent survey (Vernino and Lennon, 2004). Another case of thymoma with anti-Hu antibodies and PNS was described in a young male patient with myasthenia gravis (Simonelli et al., 2009). Three additional reports of thymic neoplasm and anti-Hu positivity have been published: one in a female patient with combined thymic epithelial tumor (consisting of small cell neuroendocrine carcinoma and thymic carcinoma) (Okamoto et al., 2011), one in a elderly female with painful distal neuropathy (Barua et al., 2012), and the latter in a 55 year old woman affected by invasive thymoma (Yang et al., 2014). Detailed clinical information was available only for 8 out of 9 individuals (Table 1). Mean age was 51.6, with female:male ratio of 6:2. As shown in Table 1, three subjects had MG, one of which with concomitant severe autonomic neuropathy characterized by intestinal pseudo-obstruction and gastrointestinal paresis. The other five did not show MG and presented heterogeneous clinical manifestations: one had neuromyelitis optica with concurrent anti-aquaporin-4 (AQP4) immunoglobulin G serum positivity, so no anti-Hu related PNS; four had sensory or painful neuropathy preceded by episodic vertigo in two cases; one of them later developed seizures, encephalitis and autonomic disturbances with constipation and fixed dilated pupils. Therefore, only the last patient, as the one described in our report, had the triad of thymoma without MG plus ANNA-1 positivity plus clinical dysautonomia; however, in this case also positive neuronal potassium channel (VGKC) autoantibodies were isolated, which could have explained part of the symptoms described (Vernino et al., 2002). In our patient, on the contrary, no other antibodies could be identified, therefore symptoms had to be ascribed only to anti-Hu positivity. Looking at CSF data, except for our case, none of the patients described so far had ANNA-1 screening done on CSF.

**Table 1:** List of published cases describing thymic neoplasms with ANNA-1 positivity.

**Article**	**Age**	**Gender**	**Clinical Presentation**	**Dysautonomia**	**Imaging**	PNS Related to ANNA-1^*^	**ANNA-1 Serum Titer**	**ANNA-1 CSF Screening**	**Muscle Ach-R Antibodies**	**Other Antibodies**	**Treatment**	**Outcome**
Vernino et al., 2002	66	F	Episodic vertigo, painful sensory neuropathy	No	MRI of the spine: normal	Yes	1920	np	Negative	VGCC/N	Two cycles of cisplatin/etoposide + subtotal thymectomy	Vertigo resolution, worsening of pain and paresthesiae, death 7 months after thymoma diagnosis
Vernino et al., 2002	34	M	MG, limbic encephalitis	No	BraIn MRI: normal	Yes	1920	np	Positive	None	preoperative plasma exchange, thymectomy and prednisone therapy (40 mg/day)	MG symptom resolution, seizure control by antiepileptics, persistence of amnesia and confusion
Vernino et al., 2002	34	F	recurrent vertigo, neuropathy, dysautonomia, encephalopathy	Constipation and fixed pupils	Brain MRIs: one non-enhancing left temporal lesion, four non-enhancing cortical lesions	Yes	960	np	Negative	VGKC	thymectomy	Seizure resolution after thymectomy
Vernino et al., 2002	39	F	MG, personality changes	No	Brain MRI: normal	Yes	960	np	Positive	CRMP-5	Pyridostigmine, corticosteroids, plasma exchange. Etoposide, cisplatin and radiation for residual thymoma	na
Simonelli et al., 2009	30	M	MG, dysautonomia, severe weight loss	Vomiting, gastroparesis, intestinal pseudo-obstruction, dry eyes and mouth and erectile failure	Brain MRI: normal	Yes	na	np	Positive	None	5 courses of plasma exchange + oral prednisone (up to a daily dose of 75 mg)	Rapid improvement of neurological symptoms (constipation, vomiting, malabsorption, and asthenia). Death few months later due to tumor relapse and severe pulmonary failure
Okamoto et al., 2011	68	F	Bilateral lower limb neuropathy	no	np	Yes	na	np	np	None	CHT with carboplatin (AUC = 4) and etoposide (100mg/m2) with concurrent RT (40 Gy)	Tumor response, but no improvement in neurologic symptoms
Barua et al., 2012	87	F	Bilateral painful tingling and numbness in lower limbs	no	np	Yes	na	np	Negative	None	Complete thymectomy	Significant improvement of peripheral neuropathy symptoms
Yang et al., 2014	55	F	Painless progressive vision loss first in the right eye and 5 months later in the left eye	no	Brain MRI: high signal intensities of both optic nerves on T2- weighted images and enhancement of the right optic nerve	No	na	np	np	AQP4-IgG	Intravenous methylprednisolone, followed by oral prednisolone (60 mg) and oral cyclosporine (600 mg) daily for 2 weeks. CHT (etoposide, paclitaxel) and prednisolone over the next 6 months. Maintenance therapy with azathioprine	Progressive deterioration in visual acuity with no light perception in the right eye and counting fingers in the left eye
Our case	71	F	Hypomimia, bradyphrenia, bradykinesia, bilateral limb rigidity with left side prevalence and mild tremor, dysautonomia	Intestinal pseudo-obstruction, acute urinary retention, dysphagia, orthostatic hypotension	Brain MRI: normal	Yes	500	Positive, present at a dilution of 1:50	Negative	None	Intravenous immunoglobulins 400 mg/kg/die for 5 days + thymectomy and RT	Complete resolution of dysautonomia, persistence of extrapyramidal symptoms

*MG, myasthenia gravis; MRI, magnetic resonance imaging; CSF, cerebrospinal fluid; CHT, chemotherapy; RT, radiotherapy; VGCC/N, voltage gated calcium channel type N; VGKC, voltage gated potassium channel; AQP4-IgG, anti-aquaporin-4 immunoglobulin G; AUC, area under curve; Gy, Gray; na, not available; np, not performed*.

* *As classified in Dalmau and Rosenfeld (2008)*.

As regards to therapy, thymectomy and/or chemotherapy (CHT)-RT were performed at the time of neurological syndrome presentation and ANNA-1 positivity in all published cases except one, where surgery and CHT-RT had been used at tumor onset and first relapse (years before the finding of positive ANNA-1) but were not repeated at the second thymoma recurrence due to patient's poor physical condition (Simonelli et al., 2009). Clinical response was variable: focusing on the two subjects who presented dysautonomia, one underwent thymectomy, but therapeutic effects of surgery on autonomic disturbances are not reported. The other one had rapid improvement of symptoms after plasma exchange plus oral prednisone. In our patient, good clinical response was obtained after the cycle of immunoglobulins, with complete and long-lasting resolution of autonomic symptoms after tumor resection.

In conclusion, the present report highlights the rarity of anti-Hu-related autonomic failure in thymoma. Furthermore, it reminds that not all dysautonomic manifestations accompanying Parkinson's disease or other parkinsonisms are to be ascribed to underlying extrapyramidal syndrome. Indeed, if symptoms have suspicious features, e.g., severe or acute-subacute presentation, and when other plausible causes for autonomic dysfunction are present, they should be specifically investigated and treated, given their good therapeutic response (plasma exchange, immunoglobulins, and/or thymectomy) and positive impact on patient's quality of life.

## Ethics statement

Written informed consent was obtained from the patient for the publication of this case report.

## Author contributions

VR: wrote the manuscript. VR, LS, MC, and EB: made table and figures. VR, BF, LS, MC, and GM: reviewed the literature. EB: performed paraneoplastic antibody testing. VR, BF, LS, MC, EB, and GM: performed final manuscript review and editing.

### Conflict of interest statement

The authors declare that the research was conducted in the absence of any commercial or financial relationships that could be construed as a potential conflict of interest.
